# MedImg: An Integrated Database for Public Medical Images

**DOI:** 10.1093/gpbjnl/qzaf068

**Published:** 2025-08-20

**Authors:** Bitao Zhong, Rui Fan, Yue Ma, Xiangwen Ji, Qinghua Cui, Chunmei Cui

**Affiliations:** Department of Biomedical Informatics, Center for Noncoding RNA Medicine, State Key Laboratory of Vascular Homeostasis and Remodeling, School of Basic Medical Sciences, Peking University, Beijing 100191, China; Department of Biomedical Informatics, Center for Noncoding RNA Medicine, State Key Laboratory of Vascular Homeostasis and Remodeling, School of Basic Medical Sciences, Peking University, Beijing 100191, China; Department of Radiology, The First Hospital of Jilin University, Changchun 130000, China; Department of Cardiology and Institute of Vascular Medicine, State Key Laboratory of Vascular Homeostasis and Remodeling, Peking University Third Hospital, Beijing 100191, China; Department of Biomedical Informatics, Center for Noncoding RNA Medicine, State Key Laboratory of Vascular Homeostasis and Remodeling, School of Basic Medical Sciences, Peking University, Beijing 100191, China; Department of Cardiology and Institute of Vascular Medicine, State Key Laboratory of Vascular Homeostasis and Remodeling, Peking University Third Hospital, Beijing 100191, China; School of Sports Medicine, Wuhan Sports University, Wuhan 430079, China; Department of Biomedical Informatics, Center for Noncoding RNA Medicine, State Key Laboratory of Vascular Homeostasis and Remodeling, School of Basic Medical Sciences, Peking University, Beijing 100191, China; School of Sports Medicine, Wuhan Sports University, Wuhan 430079, China

**Keywords:** Medical image repository, Deep learning, Computer vision, Image analysis, Multimodality

## Abstract

The advancements in deep learning algorithms for medical image analysis have garnered significant attention in recent years. While several studies have shown promising results, with models achieving or even surpassing human performance, translating these advancements into clinical practice is still accompanied by various challenges. A primary obstacle lies in the availability of large-scale, well-characterized datasets for validating the generalization of approaches. To address this challenge, we curated a diverse collection of medical image datasets from multiple public sources, containing 105 datasets and a total of 1,995,671 images. These images span 14 modalities, including X-ray, computed tomography, magnetic resonance imaging, optical coherence tomography, ultrasound, and endoscopy, and originate from 13 organs, such as the lung, brain, eye, and heart. Subsequently, we constructed an online database, MedImg, which incorporates and systematically organizes these medical images to facilitate data accessibility. MedImg serves as an intuitive and open-access platform for facilitating research in deep learning-based medical image analysis, accessible at https://www.cuilab.cn/medimg/.

## Graphical abstract

**Graphical Abstract:**
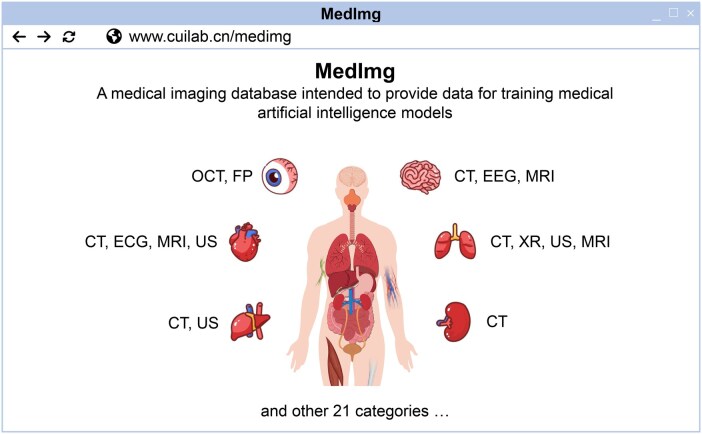

## Introduction

Medical imaging is an indispensable means to localize lesions and aid in the diagnosis and treatment of diseases [1], in which image interpretation to draw clinical conclusions is typically carried out by physicians. The computer-assisted diagnosis system emerges to expedite the diagnosis process and reduce the false positive/negative outcomes due to variations in expertise [2]. With the vast advantage of automated feature learning and extraordinary performance, deep learning techniques have become widely popular in the field of medical image analysis, including image classification for differentiating between diseased and normal individuals [3–5], organ or lesion detection to identify the small lesion region within a full image [6,7], image segmentation to partition an image into multiple segments for localization or quantification analysis [8–10], and registration for aligning more images across different modalities or time points into one coordinate system [11,12]. Numerous methods have been developed with impressive performance, particularly in image classification and segmentation. For example, Zhu et al. proposed an automatically evolutionary dense convolutional network (DenseNets) named medical image classification via ensemble bio-inspired evolutionary DenseNets (MEEDNets) [13], which outperforms other state-of-the-art methods in differentiating between acute respiratory infectious patients and non-infected individuals, as well as classifying three types of brain tumors; another model using a single convolutional neural network (CNN) for classifying skin cancer and benign nevi has demonstrated performance comparable to that of dermatologists [4]. One of the clinical applications demonstrating excellent performance is open-source artificial intelligence (AI) radiotherapy image segmentation (OSAIRIS) [14], which precisely segments the cancerous region from the healthy organ before radiotherapy and enables specialists to plan radiotherapy treatments twice as quickly. Despite the explosion of studies focused on applying deep learning algorithms to medical images, transferring these models into clinical practices remains challenging [15]. One of the primary obstacles is the scarcity of large-scale available image data, which are crucial for training, validating, and testing optimal algorithms.

Several databases gathering a wealth of medical images have been presented. The Cancer Imaging Archive (TCIA) [16] shares more than 30 million radiology images of cancers from around 37,568 subjects, organized by the National Cancer Institute (NCI). Recently, NCI Cancer Research Data Commons (CRDC) has released a new data repository, Imaging Data Commons (IDC) [17], co-locating cancer imaging collections, including TCIA, with cloud-based computing resources and data analysis tools. The Open Access Series of Imaging Studies (OASIS) [18,19] platform includes abundant neuroimaging datasets with diverse modalities, covering 3059 subjects. The Alzheimer’s Disease Neuroimaging Initiative (ADNI, http://adni.loni.usc.edu) database collects magnetic resonance imaging (MRI) and positron emission tomography (PET) images from over 1700 individuals with cognitive impairment or Alzheimer’s disease. OpenNeuro [20] enables users to openly share brain initiative data and have integrated 1066 datasets involving more than 40,000 participants across multiple modalities. Other online platforms, such as the National Institute of Mental Health Data Archive (NDA, https://nda.nih.gov/) and the Image and Data Archive (IDA) [21,22], also integrate brain-related images and support their registered users to share their research data. A national chest imaging database (NCCID) [23] comprises a diverse collection of chest images from over 7000 patients, accompanied by detailed clinical information. This database was developed to improve healthcare delivery for acute respiratory infectious patients. Furthermore, there are a fraction of AI algorithm-related competitions publishing large-scale image datasets, *e.g.*, musculoskeletal radiographs (MURA) containing 40,561 multi-view radiographic X-ray (XR) images from 14,863 studies and 12,173 patients [24]. Similarly, large online platforms like Kaggle (http://www.kaggle.com) and Grand Challenge (https://grand-challenge.org/) host data science competitions, which facilitate the development of AI algorithms and store a wealth of medical image datasets. Notably, Grand Challenge features various challenges specifically addressing medical problems, making it a valuable resource for researchers accessing medical image datasets. However, we observed that most of these datasets or databases primarily focus on individual organs/diseases, or single imaging modalities, which hinders the advancement of generalized deep learning models. It is quite necessary to develop a comprehensive and specialized platform encompassing a wide range of medical images from diverse modalities, organs, and geographic areas.

For this purpose, we proposed MedImg, an online medical image database that integrates diverse medical image datasets from multiple public sources. MedImg organizes all available data by organ and imaging modality, allowing users to easily browse, retrieve, and download all images. Moreover, for each dataset, the platform provides detailed information and sample images for preview. The MedImg online database can be freely accessed at https://www.cuilab.cn/medimg/.

## Data collection and overview

### Data collection and integration

This work aims to provide a comprehensive collection of medical images to support advancements in deep learning-based medical image analysis. Considering data privacy and ethics concerns, we utilized the keyword of “medical image” to search for publicly licensed medical image datasets. Besides, datasets that only contain scalar data are excluded. Ultimately, 105 datasets meet the inclusion criterion and are downloaded, primarily derived from Grand Challenge and Kaggle, platforms known for hosting AI technique-related competitions. Of these, 84 datasets include well-annotated labels, although not all medical image analysis tasks require annotations. In addition, these datasets were built between 2007 and 2024, with a majority established after 2015. This timeframe aligns with the surge in deep learning applications for image analysis, which, in turn, has driven the release of more medical image data. The details of all included datasets are summarized in Table S1, such as dataset name, data type, image format, modality, organ, number of image files, and deep learning task.

### Data statistics and summary

**Figure 1:**
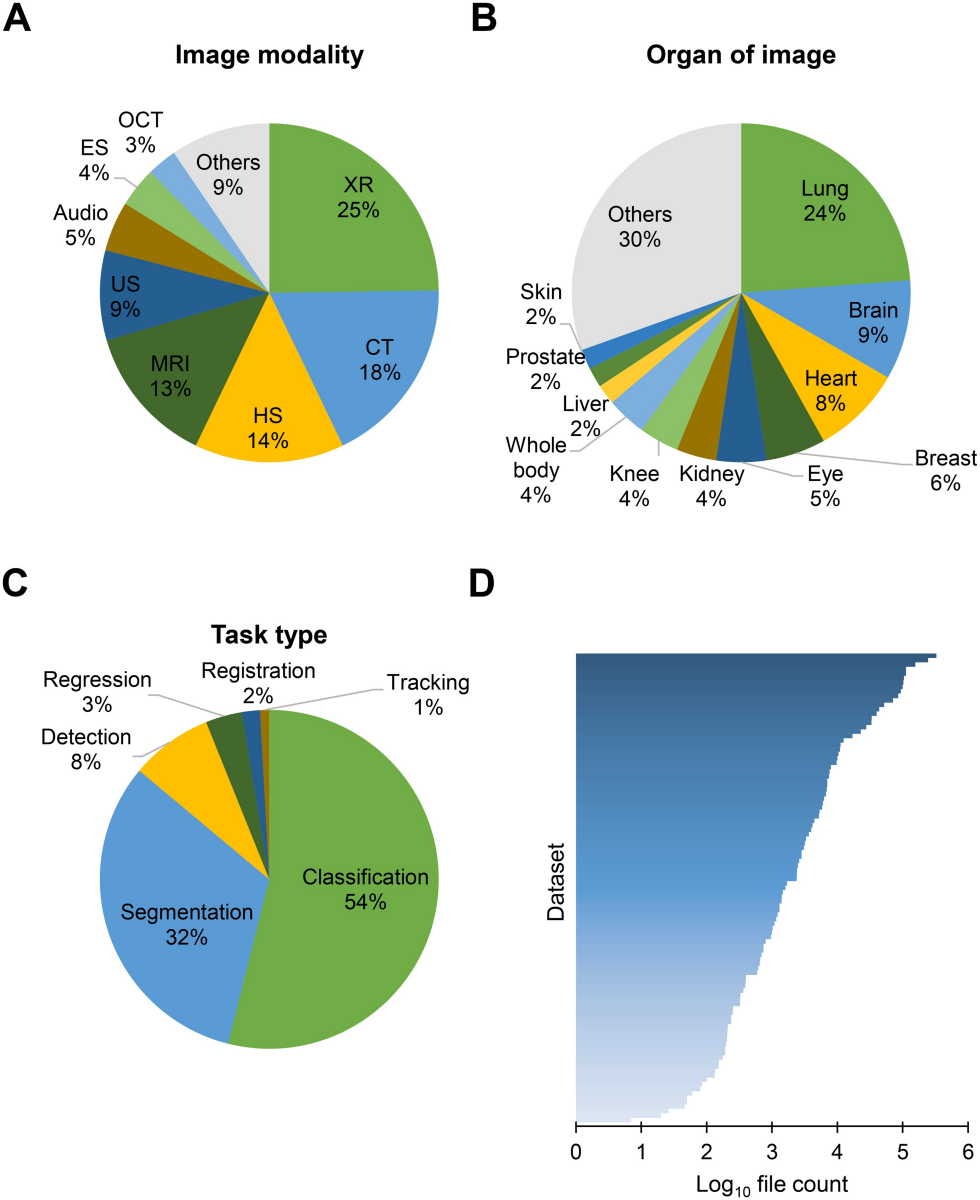
Summary of medical image datasets included in MedImg **A.** The distribution of imaging modalities of datasets. The most frequent modalities covered by these datasets are XR, CT, and MRI. **B.** The distribution of datasets across organs. The medical images in MedImg are mainly focused on the lung and brain. **C.** The distribution of analysis tasks of the medical images. Image classification and image segmentation are the most common tasks. **D.** Distribution of the number of image files per dataset. XR, X-ray; CT, computed tomography; MRI, magnetic resonance imaging; US, ultrasound; HS, histopathology slide; ES, endoscopy; OCT, optical coherence tomography.

These datasets cover various modalities, including XR, computed tomography (CT), and MRI. As shown in **Figure 1**A, XR is the most prevalent modality in medical image datasets, comprising one-fourth of the total data. XR offers a more cost-effective and accessible form to visualize the internal structure of the body compared to CT and MRI. Other common types of medical images, such as histopathology slide (HS), ultrasound (US), endoscopy (ES), optical coherence tomography (OCT), and electrocardiography (ECG), are also represented. Moreover, video and audio data of continuous disease monitoring are included as well. Figure 1B shows that the lung, brain, breast, kidney, liver, prostate, and skin are the primary organs of focus in medical image analysis. These organs are frequently affected by complicated precancerous conditions and cancers, which represent a leading cause of death [25], and as such, motivate the growth of cancer-related images involving all body parts. On the other hand, numerous datasets containing images of the heart, eye, and knee are also available, since imaging technologies are the primary means of lesion detection for these organs. **Table 1** provides an overview of datasets from various modalities for each organ. It can be observed that the heart shows the most data modalities, including CT, ECG, MRI, and US, except for those datasets with mixed or unclear sources. Additionally, the number of image files per dataset varies significantly across modalities and organs (Figure S1). More specifically, the median number of files per dataset exceeds 10,000 for ECG and dermatology images, while video datasets show a median closer to 100. Among organs, skin datasets exhibit the highest median number of files, while liver datasets have the lowest number. For different deep learning tasks, the pipeline of preprocess, structure of model, and image annotation are totally distinct [26]. As depicted in Figure 1C, over half of the datasets are employed for the image classification task, followed by segmentation accounting for 32%, which are the two most common deep learning tasks in medical image analysis. The remaining data are designed to perform lesion detection, regression, and image registration tasks. The number of image files in different datasets ranges from 7 to 327,680, with the distribution of number of image files in each dataset shown in Figure 1D. A large dataset is essential for training an excellent deep learning model [27]. Of these datasets, 35% (37 datasets) contain over 5000 image files and 23% (24 datasets) hold more than 10,000 images. In sum, we incorporated a relatively comprehensive medical image repository with a coverage of 14 modalities, 13 organs, 1,995,671 images.

**Table 1:** Summary of datasets organized by modalities and organs

Organ	Modality	File count	No. of datasets	Format	Task
Brain	CT	5519	2	jpg, png	Classification, segmentation
	EEG	1297	1	csv	Regression
	MRI	18,499	7	jpg, nii	Classification, detection, segmentation
Breast	MRI	104,851	2	dcm	Classification, segmentation
	US	1048	2	bmp, png	Classification
	XR	2700	2	jpg, pgm	Classification
Eye	FP	3235	2	jpg, ppm	Classification
	OCT	2073	3	jpg, mat	Segmentation
Heart	CT	205	1	jpg	Classification
	ECG	50,551	2	hea	Classification
	MRI	50	1	nii	Segmentation
	US	47,098	5	avi, nii, png	Classification, segmentation
Kidney	CT	15,654	4	jpg, nii	Classification, detection, segmentation
Knee	MRI	736	1	pck	Classification
	XR	13,292	3	jpg, png	Classification
Liver	CT	50	1	mhd	Segmentation
	US	7	1	mp4	Tracking
Lung	CT	270,584	6	dcm, jpg, mhd, nii, png	Classification, detection, segmentation
	MRI	253	1	nii	Classification
	XR	620,532	18	jpg, png	Classification, detection, segmentation
Prostate	MRI	7575	2	mha, nii	Regression, segmentation
Skin	SKIN	36,182	2	jpg	Classification
Teeth	XR	3653	1	png	Classification
Whole body	CT	133,338	4	dcm, png, tif	Classification, detection, registration, regression
Others	CT	746	1	nii	Segmentation
	DEV	1205	2	jpg, png	Classification
	ES	121,874	4	jpg, tif	Segmentation
	HS	487,110	15	bmp, hdf5, jpg, png, tif	Classification, detection, registration, segmentation
	US	2839	1	png	Segmentation
	XR	6629	2	dcm, jpg	Classification, detection
	Audio	36,201	5	hea, jpg, wav, webm	Classification
	Video	85	1	avi	Regression

*Note*: CT, computed tomography; EEG, electroencephalogram; MRI, magnetic resonance imaging; US, ultrasound; XR, X-ray; OCT, optical coherence tomography; ECG, electrocardiography; DEV, device; ES, endoscopy; HS, histopathology slide; FP, fundus photography.

## Database implementation and utility

In order to provide researchers with an intuitive and efficient manner to access all the data, we established an online database, which stores and organizes all medical images in a hierarchical structure. This allows users to quickly browse, search, and download images. The MedImg database (https://www.cuilab.cn/medimg/) is deployed based on Apache Tomcat server. The front end is implemented with Hypertext markup language 5 (HTML5) and Cascading style sheets level 3 (CSS3); the interactive function and visualization are implemented with jQuery; and the back end is powered by Python Django framework. In addition, we implemented a regular data update checking mechanism to keep up with the updates of the included datasets. The MedImg database can be accessed from multiple devices such as personal computer and mobile phones without registration.

**Figure 2:**
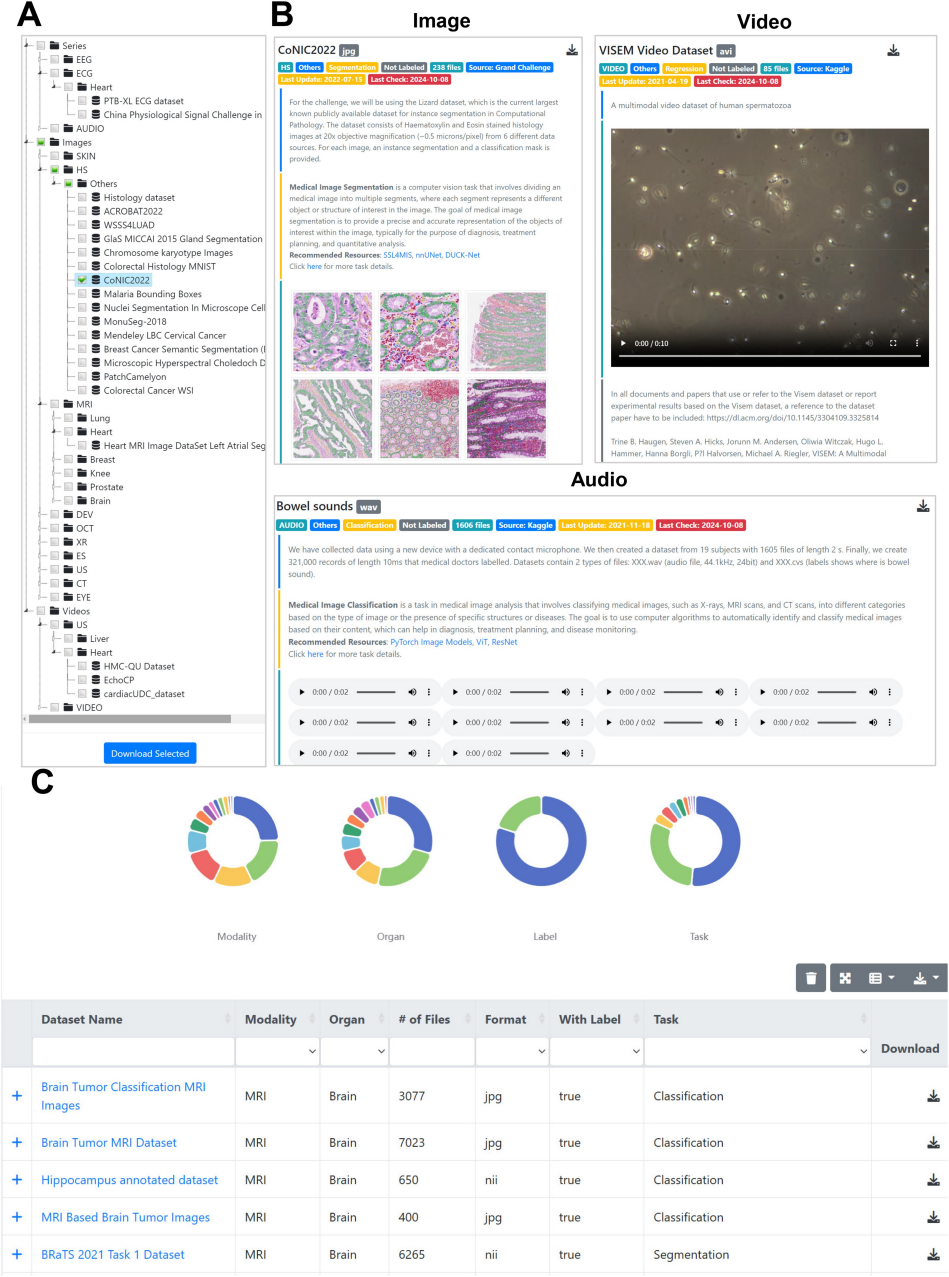
Overview of the MedImg online database **A.** Navigation tree of the “Browse” page. Users can download an individual dataset or a class of datasets via clicking on the “Download Selected” button. **B.** The details and preview for different types of datasets. The details per dataset includes modality, analysis task, source, last updated date, and number of files. **C.** The main modules of “Search” interface in MedImg. Users can input a keyword or filter by multiple fields, including modality, organ, data type, and analysis task. Meanwhile, users can click on the pie chart segments to access datasets belonging to specific categories.

The MedImg online database features four main pages, including “Home”, “Search”, “Browse”, and “Download” pages. A brief introduction related to MedImg and its update information are contained in the “Home” page. The navigation tree on the left side of the “Browse” page is organized by data type (*i.e.*, images, videos, and series), medical imaging modality (CT, MRI, OCT, *etc*.), and organ successively, as illustrated in **Figure 2**A. The checkboxes in the front of navigation terms enable users to batch obtain certain types of datasets. When the user clicks on a leaf of the navigation tree, the right side of this page presents the details and several representative samples of the corresponding dataset (Figure 2B). The detailed information includes a brief introduction for this dataset, the data format, sample size, organ, source, status of annotation, and last updated date. Moreover, we offer users access to various relevant open-source deep learning codes compatible with the current dataset. Figure 2B exhibits the preview results of different datasets in image, audio, and video formats, respectively. Moreover, this dataset can be downloaded from either the detail panel or by clicking “Download Selected” in the left navigation area. The “Search” page enables users to quickly find medical images of interest (Figure 2C). Users can directly input a keyword in the input box of “Dataset Name”. With an advanced search, users can retrieve the datasets based on multiple criteria, including the modalities of images, organ source of images, data format, labeled or unlabeled data, and the task of medical image analysis. The real-time response retrieval result is displayed at the bottom of the current page. Users can access the detail of this dataset by clicking on the dataset name link. It is noteworthy that users can separately download individual dataset or datasets of a specific class of their demand. Definitely, the online database allows users to download all data directly from the “Download” page, although this might take some time due to the large size of the data. The functionality of MedImg online database has been summarized in Table S2.

## Concluding remarks and outlook

Large-scale and well-annotated datasets are fundamental to the advancement of deep learning methods in medical image analysis. It is difficult to assess an algorithm’s generalization when using limited data, especially if it originates from a single community. Despite the high cost of manually labeling image data, institutions and researchers increasingly recognize the importance of well-characterized datasets for improving deep learning algorithms and have begun to publish and share their data. To address the need for accessible and diverse resources, we have presented a comprehensive online database, MedImg, which houses medical images from various body parts and modalities. This open-access, user-friendly platform contains 105 datasets, encompassing more than 1.9 million images, and serves as a valuable resource for researchers to obtain benchmark datasets. We anticipate that MedImg will contribute to the development of more generalized and robust deep learning-based algorithms for medical image analysis.

Undoubtedly, there are still several limitations. First, it is short of clinical information in current database, which is beneficial to the accuracy of AI-based diagnosis. Second, manual curation and normalization of all datasets using a standard protocol are necessary for simplifying the preprocessing step for users. We will continue to expand the MedImg database by incorporating new medical image datasets.

## Supplementary Material

qzaf068_Supplementary_Data

## Data Availability

The MedImg database is freely available at https://www.cuilab.cn/medimg. It has been submitted to Database Commons [28] at the National Genomics Data Center (NGDC), China National Center for Bioinformation (CNCB), which is publicly accessible at https://ngdc.cncb.ac.cn/databasecommons/database/id/10214.

## CRediT author statement

**Bitao Zhong:** Data curation, Investigation, Methodology, Validation, Writing – original draft. **Rui Fan:** Methodology, Formal analysis, Software, Validation, Visualization. **Yue Ma:** Formal analysis, Resources, Validation. **Xiangwen Ji:** Investigation, Resources. **Qinghua Cui:** Conceptualization, Supervision, Writing – review & editing, Funding acquisition. **Chunmei Cui:** Conceptualization, Supervision, Writing – original draft, Writing – review & editing, Funding acquisition. All authors have read and approved the final manuscript.
