# Supplementary material for: MedImg: An Integrated Database for Public Medical Images
Source: Genomics Proteomics Bioinformatics. 2025 Aug 20;23(4):qzaf068. doi: 10.1093/gpbjnl/qzaf068 (PMC12558383; doi:10.1093/gpbjnl/qzaf068)
Supplement: qzaf068_Supplementary_Data [file qzaf068_supplementary_data.zip › Table S2.docx]

**Table S2 Summary of the functionality of the MedImg online database**

| **Functionality** | **Details** | **Supplementary information** |
| --- | --- | --- |
| Search | By keywords | / |
|  | By modality | / |
|  | By organ | / |
|  | By deep learning task | / |
|  | By filetype | / |
|  | Download filtered datasets | / |
| Browse | Browsing data according to the hierarchical structure | / |
|  | Detail view of each dataset | A brief introduction |
|  |  | Recommend deep learning frameworks for this dataset |
|  |  | Thumbnail of this dataset |
|  |  | References/Sources of this dataset |
|  | Download selected datasets | / |
| Download | Obtaining all datasets in MedImg | / |
