# Supplementary material for: MedImg: An Integrated Database for Public Medical Images
Source: Genomics Proteomics Bioinformatics. 2025 Aug 20;23(4):qzaf068. doi: 10.1093/gpbjnl/qzaf068 (PMC12558383; doi:10.1093/gpbjnl/qzaf068)
Supplement: qzaf068_Supplementary_Data [file qzaf068_supplementary_data.zip › supplementary material captions.docx]

**Supplementary material**

**Figure S1 Distribution of number of image files per dataset across modalities and organs**

Each dot represents an individual dataset. The boxplot displays the minimum, median, and maximum number of image files, while the violin plot illustrates the distribution of file counts. The dashed line indicates the average number of image files per dataset, and modalities/organs are sorted by their average number. The last few modalities/organs are marked with “—” as they contain only one dataset. FP, fundus photography; ECG, electrocardiography; EEG, electroencephalogram; SD, standard deviation.

**Table S1 The details of all datasets included in MedImg**

**Table S2 Summary of the functionality of the MedImg online database**
