# Supplementary figures and images for: MedImg: An Integrated Database for Public Medical Images
Source: Genomics Proteomics Bioinformatics. 2025 Aug 20;23(4):qzaf068. doi: 10.1093/gpbjnl/qzaf068 (PMC12558383; doi:10.1093/gpbjnl/qzaf068)

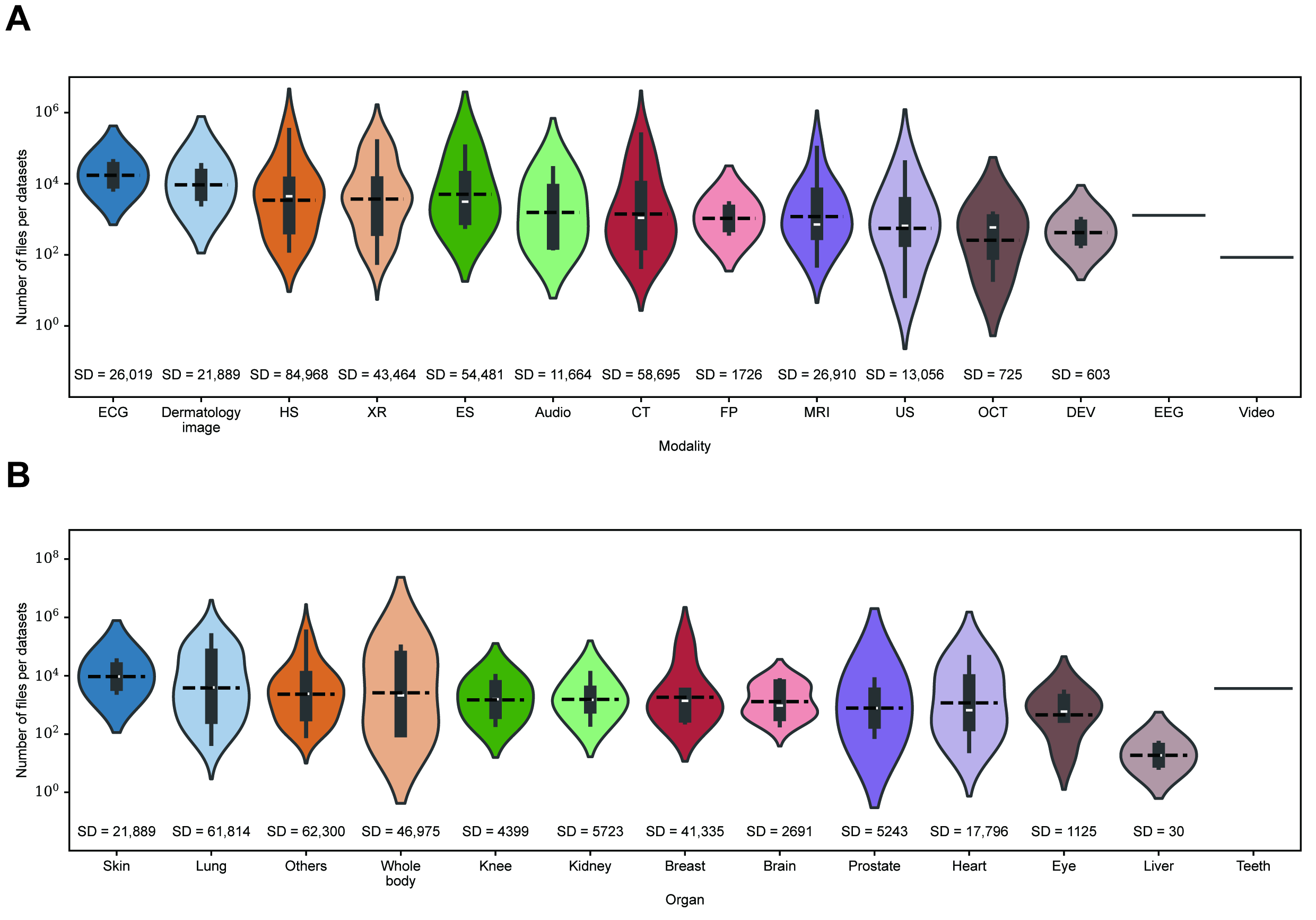

Supplement: qzaf068_Supplementary_Data [file qzaf068_supplementary_data.zip › Figure S1.tif]
